# Changes in the *Subdoligranulum* genus in patients with autoimmune disease: a systematic review and meta-analysis

**DOI:** 10.3389/fimmu.2025.1619160

**Published:** 2025-08-07

**Authors:** Lu Shen, Ying Zhao, Shuting Liu, Shangfeng Li, Qian Li, Tao-Hsin Tung, Bo Shen

**Affiliations:** ^1^ Department of Clinical Laboratory, Taizhou Hospital of Zhejiang Province Affiliated to Wenzhou Medical University, Linhai, Zhejiang, China; ^2^ School of Laboratory Medicine and Life Sciences, Wenzhou Medical University, Wenzhou, Zhejiang, China; ^3^ Department of Clinical Laboratory, Taizhou Hospital of Zhejiang Province Affiliated to Zhejiang University, Linhai, Zhejiang, China; ^4^ Evidence-based Medicine Center, Taizhou Hospital of Zhejiang Province Affiliated to Wenzhou Medical University, Linhai, Zhejiang, China

**Keywords:** autoimmune disease, subdoligranulum, inflammation-related biomarker, systematic review, meta-analysis

## Abstract

**Background:**

Autoimmune diseases have different pathogenic mechanisms but share underlying patterns of gut microbiome perturbation and intestinal barrier dysfunction. Recent evidence suggests that an arthritogenic strain of *Subdoligranulum* causes a local inflammatory response in the gut. Therefore, the aim of this review was to systematically summarize the relationships between *Subdoligranulum* and multiple autoimmune diseases.

**Objective:**

To evaluate the changes of *Subdoligranulum* in different autoimmune diseases.

**Methods:**

Four databases, including PubMed, Cochrane, Web of Science, and Embase, were searched up to June 17, 2025, to identify studies that detected *Subdoligranulum* in autoimmune diseases. A meta-analysis was conducted to compare the differences in *Subdoligranulum* between healthy people and patients with autoimmune diseases, and the changes in these bacteria under different treatments were compared for similar diseases. The relationships between *Subdoligranulum* and inflammation-related biomarkers were also analyzed.

**Study selection:**

We included articles that addressed both autoimmune diseases without intervention and the detection of *Subdoligranulum* in feces, and we presented a description of changes in bacteria in patients and healthy controls.

**Quality assessment:**

We used the Newcastle–Ottawa Scale (NOS) to independently assess the methodological quality of the case–control studies. The Journal of Biomedical Informatics (JBI) critical appraisal checklists were utilized to assess the quality and risk of bias in cross-sectional studies.

**Results:**

Twelve studies were included. These studies were conducted in four different countries and included a total of 1,792 participants (patients with autoimmune disease and healthy controls). Our meta-analysis results indicate that, compared with healthy controls, most patients with autoimmune diseases included in the study had lower levels of *Subdoligranulum* (*p* = 0.027). In addition, it was found that bacteria were associated with several inflammation-related biomarkers. For example, bacterial levels were positively correlated with C-reactive protein (CRP), lipopolysaccharide (LPS)-binding protein (LBP), and Treg cells. However, the levels were negatively correlated with IL-8. These relationships may underlie both the occurrence and development of autoimmune diseases.

**Conclusion:**

The abundance of *Subdoligranulum* in patients with organ-specific autoimmune diseases was decreased, whereas no consistent findings were observed for systemic autoimmune diseases.

**Systematic review registration:**

https://www.crd.york.ac.uk/PROSPERO/view/CRD42024543767, identifier CRD42024543767.

## 1 Introduction

Autoimmune diseases are a heterogeneous group of diseases that include more than 100 diseases, such as rheumatoid arthritis (RA), Behçet’s syndrome (BS), and Sjögren’s syndrome (SS), and affect up to 10% of the population (1). The exact pathogenesis is still unknown, but an increasing number of studies have suggested that these diseases are caused by complex gene–environment interactions, and evidence concerning the role of the environment in autoimmune diseases is growing (2).

In recent years, the increasing incidence of many diseases has been related to abnormalities in the microbial community, such that the composition and metabolomic function of microorganisms have been affected and altered, which may have beneficial, neutral, or harmful consequences for the host (3, 27). *Subdoligranulum* belongs to the Ruminococcaceae family and is an important and relatively specific producer of butyrate in the intestinal tract (4). Owing to its prevalence among healthy individuals and its unique metabolic characteristics, it has become one of the key bacterial genera in the study of the interaction between microorganisms and host immunity. A large number of gut microbes closely related to host health can produce short-chain fatty acids. Butyrate is the main energy source for colon cells and plays a core role in maintaining the integrity of the intestinal barrier, inhibiting excessive inflammatory responses, and inducing immune tolerance through various mechanisms (5).

A relevant study reported that in chronic spontaneous urticaria (CSD) and symptomatic dermographism, a decrease in these bacteria may reduce the number of Treg cells, which results in a lack of inhibition of the differentiation process of naive T cells into Th2 cells. The number of Th2 cells increases, which may promote the secretion of IgE, activate mast cells, and participate in the occurrence of this disease (6). However, new evidence in animal models suggests that a bacterial strain called *Subdoligranulum didolesgii* from an individual may cause joint inflammation (7). This seemingly contradictory phenomenon, in which the same genus of bacteria may exhibit different pro- or anti-inflammatory effects in different disease states, highlights the complexity and context-dependent role of *Subdoligranulum* in the regulation of immune function and emphasizes the necessity of in-depth research on this phenomenon.

To the best of our knowledge, although the relationship between *Subdoligranulum* and various autoimmune diseases has been described in recent years, there has been no comprehensive systematic review of autoimmune diseases and changes in these bacteria. Therefore, the purpose of this study was to provide a systematic review of the research on the variation of *Subdoligranulum* and explore the relationships between autoimmune diseases and *Subdoligranulum*.

## 2 Materials and methods

This systematic review was conducted and reported in accordance with Preferred Reporting Items for Systematic Reviews and Meta-Analyses (PRISMA) (2020) (8) guidelines, and the review program was registered with Prospective Register of Systematic Reviews (PROSPERO) (PROSPERO: CRD42024543767).

### 2.1 Search strategy

We searched four English-language databases (PubMed, Cochrane, Web of Science, and Embase) for relevant studies published and gray literature sources from the inception of the databases to June 17, 2025. The retrieval process of each database is detailed in **Supplementary Material 1**.

### 2.2 Inclusion and exclusion criteria

We included studies that met the following criteria: 1) the research was original and included clear abstract, introduction, methods, results, discussion, and conclusion; 2) the study contained a case group and control group, where the case group included patients with autoimmune disease and the control group was a healthy control; 3) the findings included changes in *Subdoligranulum* and the relationship between these bacteria and inflammation-related biomarkers; and 4) the participants were at least 18 years old. We excluded studies meeting the following criteria: 1) other types of research, such as case reports, reviews, and conference abstracts; 2) review articles and meta-analyses; and 3) incomplete data or whose full text could not be obtained. Two authors independently screened the included studies, evaluated the quality of inclusion, extracted the information, and calculated the κ value simultaneously. Any differences were resolved through discussions with the third author (Shen Bo).

### 2.3 Data extraction

The data extracted from each eligible study included the first author’s last name, year of publication, country, specific diseases associated with autoimmune diseases, study design, study subjects, changes in *Subdoligranulum*, and changes in inflammation-related biomarkers associated with *Subdoligranulum*.

### 2.4 Quality assessment

Two investigators evaluated the quality of the research (κ = 0.636). The Newcastle–Ottawa Scale (NOS) was utilized to assess the methodological quality of case–control studies (9). The assessment tool consists of eight items and rates studies from low to high quality on a scale of 0 to 9. A score of 0 to 3 indicates low methodological quality, a score of 4 to 6 indicates moderate quality, and a score of 7 to 9 indicates high methodological quality.

The JBI critical appraisal checklists were utilized to assess the quality and risk of bias in cross-sectional studies. The assessment tool consists of eight questions, and each question has three options of “yes”, “no”, and “not clear”, which account for 1, 0, and 0.5 points, respectively. After the scores obtained for each question were summed, studies were considered to have a high risk of bias and low quality if the total score was less than 3 points, moderate risk of bias and moderate quality if the total score was 3 to <6 points, and low risk of bias and high quality if the total score was 6–8 points.

### 2.5 Meta-analysis

Meta-analysis was performed on differences in the abundance of *Subdoligranulum* between patients with autoimmune diseases and the control group in studies with sufficiently reported data. Statistical heterogeneity between studies was assessed for each outcome by examining study-specific effect sizes and heterogeneity (I^2^) statistics. In meta-analyses of multiple studies for a specific outcome, a fixed-effects estimate was calculated if the I^2^ value was <50%, and a random-effects estimate was calculated if the I^2^ value was ≥50%. For studies without accurate data, estimations on the basis of the visualized graphs were made. Therefore, the results of our meta-analysis may not be highly accurate.

Statistical analysis was performed using the STATA software (Version 18.0, StataCorp, College Station, TX, USA). Statistical significance was set at *p* < 0.05.

## 3 Results

### 3.1 Study selection

**Figure 1:**
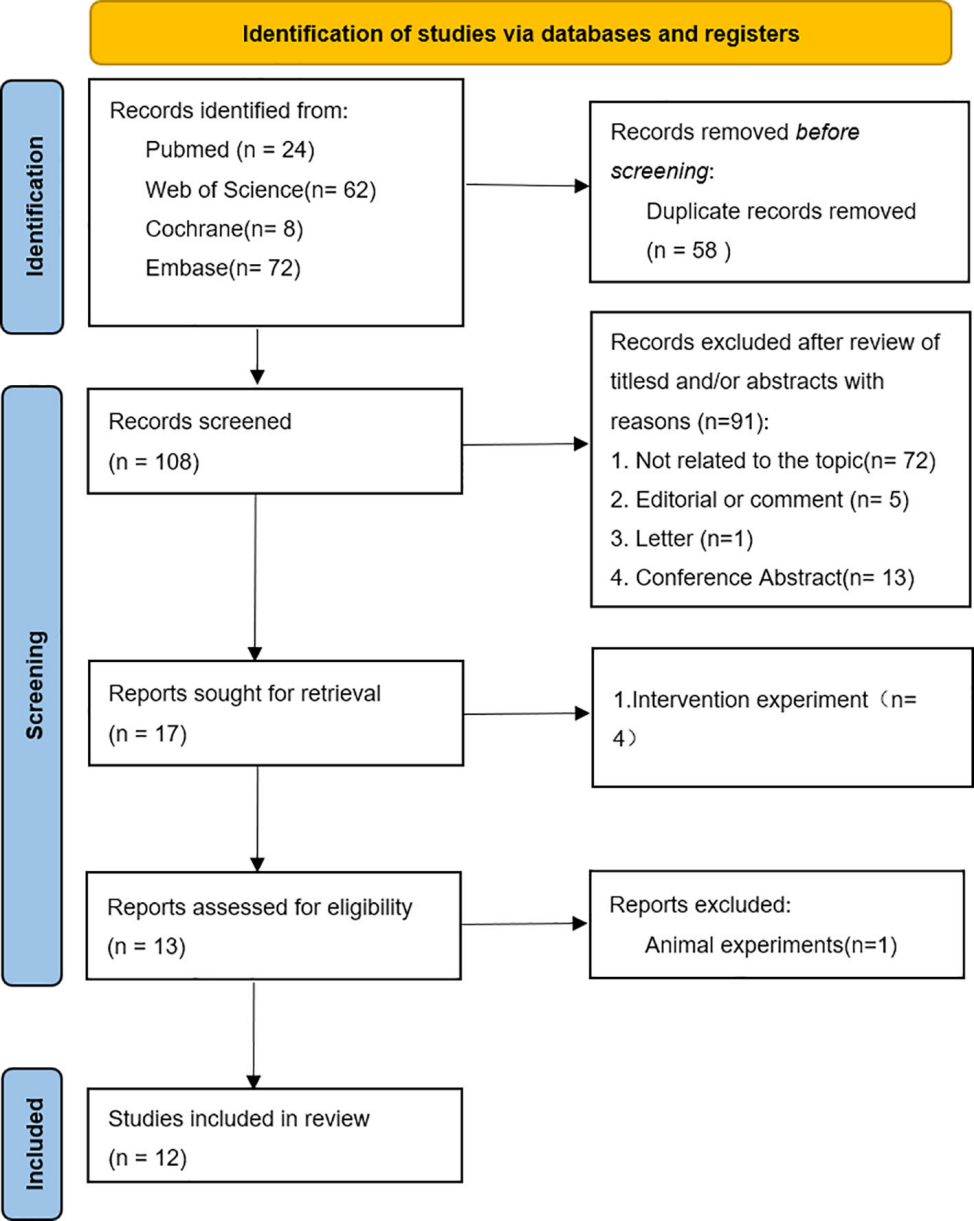
PRISMA study flowchart.

A total of 166 articles were retrieved from PubMed, Cochrane, Web of Science, and Embase. After the exclusion of 58 duplicate articles, 91 articles were excluded on the basis of title and abstract in the identification stage, and the full texts of the remaining 17 articles were checked. Finally, on the basis of the study objectives and inclusion or exclusion criteria, a total of 12 studies were deemed eligible for inclusion in this systematic review. The flowchart of the research selection process is shown in **Figure 1**.

### 3.2 Study characteristics

All included studies were observational studies, two were cross-sectional studies, and 10 were case–control studies. The features of the included studies and the main outcomes are shown in **Table 1**. Most of these studies (n = 10) were published in the last 5 years (2021–2025), with a total of eight studies conducted in China, two in Europe, and two in Korea.

**Table 1:** Characteristics of the 12 studies included in the systematic review.

Author, year	Country	Disease	Study design	Study subjects	*Subdoligranulum* changes (patients vs. healthy controls)	Inflammation-related biomarkers associated with *Subdoligranulum*
Consolandi et al., 2015 (10)	Italy	BS	Case–control study	22 BS, 16 HC	Abundance change: BS: 1.93% ± 1.75% HC: 3.28% ± 2.20% *p* = 0.049	–
Liu et al., 2021 (6)	China	CSD	Case–control study	25 CSD, 25 HC	LDA scores (log10): CSD < 2 HC > 2 *p* < 0.05	Treg cell ↓
Yu et al., 2021 (11)	China	MPA	Case–control study	71 MPA [35 aMPA and 36 inMPA] and 34 HC	aMPA vs. HC: ↓ (*p* = 0.01) inMPA vs. HC: ↓ (*p* = 0.002)	–
Moon et al., 2020 (12)	Korea	SS, DES	Case–control study	10 SS, 14 DES, and 12 HC	SS vs. HC: no significant difference DES vs. HC: ↓ (*p* = 0.035)	–
Liu et al., 2023 (13)	China	Hashimoto’s thyroiditis	Cross-sectional study	18 SH, 30 EU, and 28 HC	EU vs. HC: no significant difference SH vs. HC: ↓ (*p* < 0.05)	A negative correlation between LPB and *Subdoligranulum*
Shi et al., 2021 (14)	China	GD, GO	Case–control study	30 GD, 33 GO, and 32 HC	GD vs. HC: ↓ (*p* < 0.05) GO vs. HC: no significant difference	–
Jeong et al., 2024 (15)	Korea	GD	Case–control study	29 GD and 130 HC	GD vs. HC: NO significant difference	–
De Groot et al., 2017 (16)	Netherlands	T1D	Case–control study	53 T1D and 50 HC	Abundance change: T1D: 0.0068 HC: 0.0282 *p* = 0.764	LBP, *r* = 0.519, *p* < 0.001 CRP, *r* = 0.362, *p* < 0.05
Liu et al., 2020 (17)	China	HT	Cross-sectional study	45 HTN, 18 HTH, and 34 HC	LDA scores (log10): HTH > 3 HC < 2 *p* < 0.05	–
Yao et al., 2022 (18)	China	SLE	Case–control study	21 SLE-d, 17 SLE-nd, and 32 HC	SLE-d vs. HC: ↓ (*p* = 0.047) SLE-nd vs. HC: ↑ (LDA: 4.22, *p* = 0.002)	IL-2, IL-6: no significant difference
Wang et al., 2025 (19)	China	RA	Cross-sectional study	262 RA and 475 HC	RA vs. HC: ↓(*p* < 0.05)	–
Song et al., 2023 (20)	China	SS	Cross-sectional study	101 SS and 101 HC	SS vs. HC: ↑ (*p* < 0.001)	–

BS, Behçet’s syndrome; CSD, chronic spontaneous urticaria and symptomatic dermographism; T1D, type 1 diabetes; SS, Sjogren’s syndrome; DES, dry eye syndrome; MPA, microscopic polyangiitis; aMPA, microscopic polyangiitis at incipient active stage; inMPA, microscopic polyangiitis at remissive stage; SH, Hashimoto’s thyroiditis with subclinical hypothyroidism; EU, Hashimoto’s thyroiditis with euthyroidism; GD, Graves’ disease; GO, Graves’ orbitopathy; HT, Hashimoto’s thyroiditis; HTN, Hashimoto’s thyroiditis with euthyroidism; HTH, Hashimoto’s thyroiditis with hypothyroidism; SLE, systemic lupus erythematosus; SLE-d, systemic lupus erythematosus with depression; SLE-nd; systemic lupus erythematosus without depression; RA, rheumatoid arthritis; IL, interleukin; CRP, C-reactive protein; Treg cell, T regulatory cells; LBP, lipopolysaccharide-binding protein; HC, healthy control; ↑, Increase; ↓, Decrease.

Although the methods used to describe the changes in *Subdoligranulum* differed, the difference in bacteria between patients and healthy controls was mentioned in all included studies. However, only five of these studies assessed the relationship between bacteria and some inflammation-related biomarkers.

The results of the methodological quality assessment of the NOS and JBI tools are summarized in **Tables 2**, **3**, respectively. In most studies, the definitions and selection methods of case groups were well illustrated. However, only one case–control study controlled for major or minor confounders in the case and control groups. Overall, the included studies were of moderate quality.

**Table 2:** Critical appraisal of the included case–control studies (NOS).

Study	Selection				Comparability		Exposure			Total Score
	Q1	Q2	Q3	Q4	Q5	Q6	Q7	Q8	Q9	
Consolandi et al., 2015 (10)	★	★	★	NR	★	NR	★	★	NR	6
Liu et al., 2021 (6)	★	★	★	NR	NR	★	★	★	NR	6
Yu et al., 2021 (11)	★	★	★	NR	NR	NR	★	★	NR	5
Moon et al., 2020 (12)	★	★	★	★	NR	NR	★	★	NR	6
Shi et al., 2021 (14)	★	★	★	NR	NR	NR	★	★	NR	5
Jeong et al., 2024 (15)	★	★	**★**	★	NR	NR	★	★	NR	6
De Groot et al., 2017 (16)	★	★	★	NR	NR	★	★	★	NR	6
Yao et al., 2022 (18)	★	★	★	NR	NR	★	★	★	NR	6

Q1, Is the case definition adequate? Q2, Representativeness of the cases; Q3, Selection of controls; Q4, Definition of controls; Q5, Study controls for the most important factor; Q6, Study controls for the second important factor; Q7, Was the measurement method of *Subdoligranulum* described? Q8, Were the methods of measurement similar for cases and controls? Q9, Non-response rate.

★, Yes; NR, not reported; NOS, Newcastle-Ottawa Scale.

**Table 3:** Critical appraisal of the included cross-sectional studies (JBI critical appraisal for cross-sectional studies).

Study	Q1	Q2	Q3	Q4	Q5	Q6	Q7	Q8	Total
Liu et al., 2023 (13)	Y	Y	Y	Y	N	N	Y	Y	6
Liu et al., 2020 (17)	Y	Y	Y	Y	N	N	Y	Y	6
Wang et al., 2025 (19)	Y	Y	Y	Y	N	N	Y	Y	6
Song et al., 2023 (20)	Y	N	Y	Y	N	N	Y	Y	5

(Q1) Were the inclusion criteria for study subjects clear? (Q2) Were the research objects and places described in detail? (Q3) Was the assessment method of exposure factors valid and credible? (Q4) Was the assessment method of health problems objective and standard? (Q5) Were the confounding factors clearly defined? (Q6) Were there any measures to control confounders? (Q7) Were the outcomes measured in a valid and credible way? (Q8) Was the statistical analysis appropriate?

### 3.3 Patient characteristics

Liu et al. reported that the median age was 35 years (30–40 years) for patients with Hashimoto’s thyroiditis with euthyroidism (EU)–, 38 years (30–51 years) for patients with Hashimoto’s thyroiditis with subclinical hypothyroidism (SH)–, and 30 years (26.5–39.5 years) for the control group (*p* = 0.621). The EU, SH, and control groups included 27 (90%), 17 (94%), and 24 (86%) female subjects (*p* = NA), respectively (13). Shi et al. reported that the median age was 46 years (34.3–57.7 years) for patients with Graves’ orbitopathy (GO), 45 years (32.2–57.8 years) for patients with Graves’ disease (GD), and 43.4 years (33.7–53.1 years) for the control group (*p* = NS). The GO, GD, and control groups included 16 (48%), 20 (67%), and 16 (50%) female subjects (*p* = NA), respectively (14). Jeong et al. reported that the median age of patients with GD was 45.7 years (20–68 years). However, the age of the healthy control group was not mentioned. The GD and control groups included 15 (52%) and 120 (52%) female subjects (*p* = NS), respectively. De Groot et al. reported that the median age was 35 years (26–44 years) for patients with type 1 diabetes (T1D)– and 36 years (23–49 years) for the control group – (*p* = NS). The T1D and control groups included 25 (47%) and 24 (48%) female subjects (*p* = NS), respectively (16). Liu et al. reported that the median age was 34.6 years (33.6–35.6 years) for patients with Hashimoto’s thyroiditis with euthyroidism (HTN), 36.3 years (34.2–38.4 years) for patients with Hashimoto’s thyroiditis with hypothyroidism (HTH), and 29.6 years (29–30.2 years) for the control group (*p* = 0.0003). The HTN, HTH, and control groups included 45 (100%), 18 (100%), and 34 (100%) female subjects (*p* = NA), respectively (17).

Consolandi et al. reported that the median age was 41.1 years for patients with BS and 43.4 years for the control group (*p* = NS). The healthy controls in the present study lived in the same house as the BS patients, which may eliminate confounding factors. In 11 cases, the control was a cohabitant, whereas in the other five cases, the control was a first-degree relative. The BS and control groups included 10 (45%) and 10 (63%) female subjects (*p* = NA), respectively (10). Liu et al. reported that the median age was 36 years (24.2–47.8 years) for patients with CSD and 36.8 years (27.1–46.5 years) for the control group (*p* = NS). The CSD and control groups included 15 (60%) and 15 (60%) female subjects (*p* = NS), respectively (6). Yu et al. reported that the median age was 61 years (55–65 years) for patients with microscopic polyangiitis with kidney involvement at the remissive stage (inMPA)–, 61 years (55–68 years) for patients with microscopic polyangiitis with kidney involvement at the incipient active stage (aMPA)–, and 58 years (53–63 years) for the control group – (*p* = NS). The inMPA, aMPA, and control groups included 36 (64%), 35 (65%), and 23 (66%) female subjects (*p* = NA), respectively (11). Moon et al. reported that the median age was 58.50 years (47–75 years) for patients with SS, 46.29 years (24–63 years) for patients with DES, and 47.50 years (22–62 years) for the control group (*p* = 0.061). The SS, dry eye syndrome (DES), and control groups included 10 (100%), 12 (86%), and 9 (75%) female subjects (*p* = 0.250), respectively (12). Yao et al. reported that the median age was 39.66 years (26.4–51.86 years) for patients with systemic lupus erythematosus with depression (SLE-d), 43.58 years (29.11–58.05 years) for patients with systemic lupus erythematosus without depression (SLE-nd), and 38.51 years (24.23–52.79 years) for the control group (*p* = 0.35). The SLE-d, SLE-nd, and control groups included 21 (100%), 17 (100%), and 32 (100%) female subjects (*p* = NA), respectively (18). Wang et al. reported that the median age was 52.5 years (37.2–67.8 years) for patients with RA and 40.6 years (29.4–51.8 years) for the control group (*p* = NA). The RA and control groups included 195 (73.4%) and 273 (57.5%) female subjects (*p* = NA), respectively (19). Song et al. reported that the median age was 56 years (20–84 years) for patients with SS– and 50 years (23–77 years) for the control group – (*p* = NS). The SS and control groups included 96 (95.0%) and 94 (93.1%) female subjects (*p* = NS), respectively (20).

### 3.4 Organ-specific autoimmune diseases

Six of the diseases involved in six of the 12 studies included were organ-specific autoimmune diseases. We compared the EU group reported by Liu et al., the GO group reported by Shi et al., the GD group reported by Jeong et al., the T1D group reported by De Groot et al., and healthy controls, and we found that there were no significant differences in *Subdoligranulum* in feces from the different groups (13–16, 21). The levels of *Subdoligranulum* in feces of the SH group (*p* < 0.05) reported by Liu et al. and the GD group (*p* < 0.05) reported by Shi et al. were significantly lower than those from healthy controls (13, 14). However, the prevalence of *Subdoligranulum* in feces in HTH patients reported by Liu et al. was significantly greater than that in controls [Linear Discriminant Analysis (LDA) score (log10): HTH > 3, HC < 2, *p* < 0.05] (17). Interestingly, the relative abundance of this bacterium was greater in healthy controls. However, we found no significant differences between the EU, GO, GD, and T1D groups (13, 14, 16, 21). In addition, three of these studies reported an association between bacteria and inflammation-related biomarkers. *Subdoligranulum* was negatively correlated with lipopolysaccharide-binding protein (LBP) (13). *Subdoligranulum* was significantly correlated with LBP (*r* = 0.519, *p* < 0.001) and C-reactive protein (CRP) (*r* = 0.362, *p* < 0.05) (16). *Subdoligranulum* was negatively correlated with serum IL-8 levels (*r* = −0.95, *p* < 0.05) (21).

### 3.5 Systemic autoimmune disease

Six of the 12 studies included were about systemic autoimmune diseases. There was no significant difference in *Subdoligranulum* in feces from the SS group and the healthy control group (12). Compared with that in healthy controls, we found that the level of *Subdoligranulum* decreased significantly in the BS group as reported by Consolandi et al. (abundance change: BS: 1.93% ± 1.75%, HC: 3.28% ± 2.20%, *p* = 0.049), the CSD group as reported by Liu et al. [LDA scores (log10): CSD < 2, HC > 2, *p* < 0.05], the aMPA (*p* = 0.01) and inMPA groups (*p* = 0.002) as reported by Yu et al., the DES group as reported by Moon et al. (*p* = 0.035), the RA group as reported by Wang et al. (*p* < 0.05), and the SLE-d group (*p* = 0.047) as reported by Yao et al. (6, 10–12, 18, 19) Yao et al. (LDA: 4.22, *p* = 0.002) reported that the number of bacteria was significantly greater in patients with SLE than in controls (18). Interestingly, Moon et al. reported that even though there was no significant difference between bacteria in SS patients and healthy controls, the relative abundance of bacteria in SS patients was lower than that in healthy controls (12). Two of the five studies reported an association between bacteria and inflammation-related biomarkers. One study reported that a decrease in the abundance of *Subdoligranulum* led to a decrease in Treg cells, which promoted the Th2 response and the occurrence of CSD (6). Another study reported that there was no significant association between bacteria and IL-2 and IL-6 (18).

### 3.6 Meta-analysis

**Figure 2:**
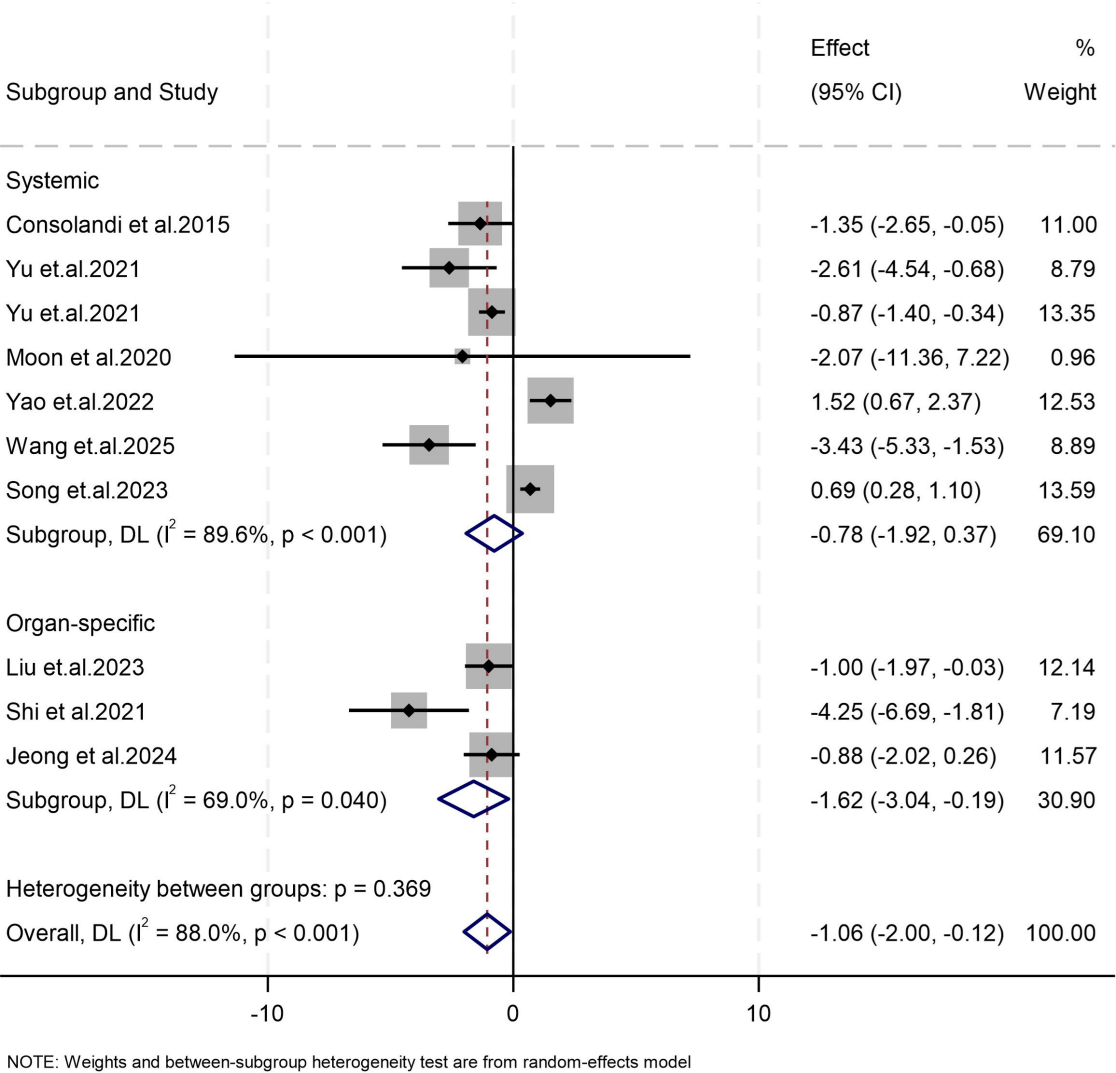
Forest plots of studies on differences in the abundance of *Subdoligranulum* between patients with autoimmune diseases and the control group. The size of the square is proportional to study-specific statistical weights, horizontal lines represent 95% confidence intervals, and diamonds represent summary measures of association.

Our meta-analysis of nine studies revealed that the abundance of *Subdoligranulum* in patients with autoimmune diseases was significantly lower than that in healthy controls (Weighted Mean Difference (WMD) = −1.06, 95% CI [−2.00, −0.12], *p* = 0.027; I^2^ = 88.0%, *p* < 0.001; **Figure 2**). Subgroup analysis was conducted on the basis of disease characteristics. Similar results were found in the organ-specific autoimmune disease subgroup (Weighted Mean Difference (WMD) = −1.62, 95% CI [−3.04, −0.19], *p* = 0.026; I^2^ = 69.0%, *p* = 0.040), whereas no significant results were observed in the systemic autoimmune disease subgroup (WMD = −0.78, 95% CI [−1.92, 0.37], *p* = 0.182; I^2^ = 89.6%, *p* < 0.001). Due to the small number of studies, it is not feasible to assess publication bias.

## 4 Discussion

To the best of our knowledge, this is the first systematic review of studies on the relationship between *Subdoligranulum* and autoimmune diseases. Our analysis revealed that most patients with autoimmune diseases have lower bacterial counts than healthy people. However, our findings do not confirm that bacteria can inhibit the development of autoimmune diseases.

To date, many studies have shown a relationship between *Subdoligranulum* bacteria and autoimmune diseases. In the included studies, patients with different autoimmune diseases had lower bacterial abundances than healthy individuals, especially those with organ-specific autoimmune diseases. The bacterium is a well-known producer of butyrate in short-chain fatty acids (22). However, the specific mechanism of action in this disease is still unclear (6). A possible mechanism between this bacterium and CSD has been described. A decrease in the number of bacteria reduces the number of Treg cells, weakens their function, and eventually activates mast cells, leading to the development of disease. However, some previous animal studies have shown that the *Subdoligranulum* variable alone can induce mice to produce Treg cells through the RORγt pathway, thereby reducing the Th2 immune response in food allergies (23). Studies have also shown that the isolation of seven bacteria from individual feces can stimulate Th17 cell proliferation in mice, deposit IgA and IgG in the joints, and induce joint swelling in mice (7). This differs from the current findings, potentially due to a different type of bacteria. *In vivo* experiments did not classify the bacteria, and animal experiments selected a stronger pathogenic type of bacteria, resulting in different results. Second, four of the studies we included mentioned specific relationships between bacteria and inflammation-related factors. We found that the findings concerning the anti-inflammatory and protective effects of bacteria were based on limited evidence. Other studies showed inconsistent results, which weakens this claim. Liu et al. (6) reported that a decrease in bacteria caused a decrease in Treg cells. Liu et al. (13) reported that bacteria were negatively correlated with LBP. Shan et al. (21) reported that bacteria were negatively correlated with IL-8. These three studies revealed that bacteria may play a role in inhibiting inflammation. De Groot et al. (16) reported that bacteria were positively correlated with CRP and LBP. The results of this study differ from those of the previous three articles, possibly because of the different diseases involved and the different pathogeneses of the diseases. The role of *Subdoligranulum* in various autoimmune diseases is inconsistent.

The median age of onset of the autoimmune diseases included in the studies assessed in this review varied greatly, ranging from childhood to old age. Ageing is associated with reduced microbial diversity, diminished short-chain fatty acid (SCFA) production, and compromised gut barrier integrity (24). Specifically, Ruminococcaceae, a key butyrate producer, decreases with age because of immunosenescence and shifts in the inflammatory milieu (25), which may exacerbate autoimmune pathogenesis in older patients. Conversely, the younger autoimmune group may retain greater subnuclear richness, which may alleviate disease progression. Future research should stratify subgroups by age to describe the role of age in autoimmunity.

In the organ-specific autoimmune disease analysis, we observed differences in *Subdoligranulum* levels compared with those in the healthy control group. These findings indicate that different autoimmune diseases share similar pathogenesis mechanisms and may act consistently through the actions of *Subdoligranulum*. However, no such difference was observed in systemic autoimmune diseases. This heterogeneity suggests that specific target organs, the extent of inflammation, and other factors may influence the composition of the gut microbiota. Given the high heterogeneity among the studies, it is necessary to conduct more research to further investigate the changes in *Subdoligranulum* in individual autoimmune diseases. Moreover, future studies should compare the differences and variations between organ-specific and systemic autoimmune diseases to clarify the effects of local inflammation and systemic inflammation on changes in the gut microbiota.

The human gut microbiome is a complex ecosystem. Among its understudied symbiotic organisms, the genus *Subdoligranulum* has recently emerged as an important taxonomic group because it is widely colonized in healthy individuals, and its abundance changes under various disease conditions. The current literature is fragmented and limited to association studies in cohort analysis, with limited understanding of its functional mechanism in microbial networks or host–microbial interactions. Advances in microbial genomics and metabolomics have revealed the ability of this bacterium to produce SCFAs, particularly butyrate, a key immunomodulatory metabolite associated with intestinal barrier integrity and anti-inflammatory responses (28). This metabolic characteristic makes the inferior neural zone a promising candidate for microbiota-targeted therapy, with the aim of restoring the probiotic community through probiotics, prebiotics, synthetic bacteria, or live biological therapeutic products (26). However, the translational potential of intervention measures centered on *Subdoligranulum* is still hindered by the knowledge gap regarding its strain-level diversity, ecological resilience, and clinical environmental safety.

This study has several limitations. First, this review ultimately included only 12 studies. Furthermore, there were almost no recurrent diseases, which leads to underrepresentation. Second, considering confounding factors such as diet and lifestyle, in one study, only the healthy controls were first-degree relatives or cohabiting. Third, the studies we included have difficulty predicting similar causal relationships between bacteria and diseases. Fourth, multiple subtypes of this bacterium have been discovered thus far, but most of the literature has not conducted specific subtype analyses. In the future, more studies on the impact of different subtypes on autoimmune diseases, especially single diseases, need to be published. Moreover, certain outcome indicators were added to observe the specific effects.

## 5 Conclusion

In conclusion, our findings indicate that the *Subdoligranulum* abundance is decreased in patients with autoimmune diseases, especially those with organ-specific autoimmune disorders. In the future, large-scale targeted research is needed to specifically discuss whether there are consistent subpopulations of microbiota alterations in organ-specific autoimmune diseases, adjust for confounding factors, and identify subpopulations related to local organs or local inflammation.

## Data Availability

The original contributions presented in the study are included in the article/**Supplementary Material**. Further inquiries can be directed to the corresponding authors.
