# Supplementary material for: Changes in the Subdoligranulum genus in patients with autoimmune disease: a systematic review and meta-analysis
Source: Front Immunol. 2025 Aug 7;16:1619160. doi: 10.3389/fimmu.2025.1619160 (PMC12367689; doi:10.3389/fimmu.2025.1619160)
Supplement: Supplementary file 1 [file DataSheet1.zip › Supplementary Material 1.docx]

|  | Search strategy | Pubmed | Embase | Cochrane | Web of science |
| --- | --- | --- | --- | --- | --- |
| #1 |  | 862,264 | 1,829,105 | 149,527 | 2,189,475 |
| #2 |  | 405 | 615 | 50 | 665 |
| #3 | #1 AND #2 | 24 | 72 | 8 | 62 |

#1=Autoimmune Diseases or Disease, Autoimmune or Diseases, Autoimmune or Autoimmune Disease or Addison Disease or Disease, Addison or Primary Adrenal Insufficiency or Adrenal Insufficiency, Primary or Primary Hypoadrenalism or Hypoadrenalism, Primary or Hypoadrenalisms, Primary or Addison's Disease or Addisons Disease or Primary Adrenocortical Insufficiency or Adrenocortical Insufficiencies, Primary or Adrenocortical Insufficiency, Primary or Insufficiencies, Primary Adrenocortical or Insufficiency, Primary Adrenocortical or Primary Adrenocortical Insufficiencies OR Anemia, Hemolytic, Autoimmune or Hemolytic Anemia, Autoimmune or Autoimmune Hemolytic Anemia or Anemia, Autoimmune Hemolytic or Autoimmune Hemolytic Anemias or Autoimmune Haemolytic Anaemia or Anaemia, Autoimmune Haemolytic or Autoimmune Haemolytic Anaemias or Haemolytic Anaemia, Autoimmune or Cold Agglutinin Disease or Agglutinin Disease, Cold or Cold Agglutinin Diseases or Cold Antibody Disease or Cold Antibody Diseases or Cold Antibody Hemolytic Anemia or Anemia, Hemolytic, Cold Antibody or Idiopathic Autoimmune Hemolytic Anemia or Acquired Autoimmune Hemolytic Anemia or Anemia, Hemolytic, Idiopathic Acquired OR Anti-Glomerular Basement Membrane Disease or Anti Glomerular Basement Membrane Disease or Anti-GBM Disease or Anti GBM Disease or Lung Purpura with Nephritis or Goodpasture's Syndrome or Goodpastures Syndrome or Syndrome, Goodpasture's or Goodpasture Syndrome or Syndrome, Goodpasture OR Anti-Neutrophil Cytoplasmic Antibody-Associated Vasculitis or Anti Neutrophil Cytoplasmic Antibody Associated Vasculitis or Pauci-Immune Vasculitis or Pauci Immune Vasculitis or Pauci-Immune Vasculitides or Vasculitides, Pauci-Immune or Vasculitis, Pauci-Immune or ANCA-Associated Vasculitis or ANCA Associated Vasculitis or Vasculitis, ANCA-Associated or ANCA-Associated Vasculitides or ANCA Associated Vasculitides or ANCA-Associated Vasculitide or Vasculitide, ANCA-Associated or Vasculitides, ANCA-Associated OR Churg-Strauss Syndrome or Churg Strauss Syndrome or Syndrome, Churg-Strauss or Allergic Granulomatous Angiitis or Allergic Granulomatous Angiitides or Angiitides, Allergic Granulomatous or Granulomatous Angiitides, Allergic or Granulomatous Angiitis, Allergic or Angiitis, Allergic Granulomatous or Vasculitis, Churg-Strauss or Churg-Strauss Vasculitis or Vasculitis, Churg Strauss or EGPA Disorder or Disorder, EGPA or Disorders, EGPA or EGPA Disorders or Allergic Angiitis and Granulomatosis or Allergic Granulomatosis or Allergic Granulomatoses or Granulomatoses, Allergic or Granulomatosis, Allergic or Allergic Granulomatous and Angiitis or Eosinophilic Granulomatous Vasculitis or Granulomatous Vasculitides, Eosinophilic or Granulomatous Vasculitis, Eosinophilic or Vasculitides, Eosinophilic Granulomatous or Vasculitis, Eosinophilic Granulomatous or Eosinophilic Granulomatosis with Polyangiitis or Allergic Angiitis or Allergic Angiitides or Angiitides, Allergic or Angiitis, Allergic or Granulomatous Allergic Angiitis or Allergic Angiitides, Granulomatous or Allergic Angiitis, Granulomatous or Angiitides, Granulomatous Allergic or Angiitis, Granulomatous Allergic or Granulomatous Allergic Angiitides OR Granulomatosis with Polyangiitis or Granulomatosis with Polyangiitides or Polyangiitides, Granulomatosis with or Polyangiitis, Granulomatosis with or with Polyangiitides, Granulomatosis or with Polyangiitis, Granulomatosis or Wegener Granulomatosis or Granulomatosis, Wegener or Granulomatosis, Wegener's or Wegener's Granulomatosis OR Microscopic Polyangiitis or Microscopic Polyangiitides or Polyangiitides, Microscopic or Polyangiitis, Microscopic OR Antiphospholipid Syndrome or Syndrome, Antiphospholipid or Hughes Syndrome or Syndrome, Hughes or Antiphospholipid Antibody Syndrome or Antibody Syndrome, Antiphospholipid or Antiphospholipid Antibody Syndromes or Syndrome, Antiphospholipid Antibody or Anti-Phospholipid Antibody Syndrome or Anti Phospholipid Antibody Syndrome or Antibody Syndrome, Anti-Phospholipid or Syndrome, Anti-Phospholipid Antibody or Anti-Phospholipid Syndrome or Anti Phospholipid Syndrome or Syndrome, Anti-Phospholipid OR Arthritis, Juvenile or Juvenile Arthritis or Childhood Arthritis or Arthritides, Childhood or Arthritis, Childhood or Childhood Arthritides or Arthritis, Juvenile Chronic or Juvenile Chronic Arthritis or Chronic Arthritis, Juvenile or Juvenile Idiopathic Arthritis or Idiopathic Arthritis, Juvenile or Arthritis, Juvenile Idiopathic or Juvenile Rheumatoid Arthritis or Arthritis, Juvenile Rheumatoid or Rheumatoid Arthritis, Juvenile or Oligoarthritis, Juvenile or Juvenile Oligoarthritis or Psoriatic Arthritis, Juvenile or Arthritis, Juvenile Psoriatic or Juvenile Psoriatic Arthritis or Enthesitis-Related Arthritis, Juvenile or Arthritis, Juvenile Enthesitis-Related or Enthesitis Related Arthritis, Juvenile or Juvenile Enthesitis-Related Arthritis or Polyarthritis, Juvenile, Rheumatoid Factor Negative or Juvenile-Onset Still Disease or Juvenile Onset Still Disease or Still Disease, Juvenile-Onset or Still Disease, Juvenile Onset or Still's Disease, Juvenile-Onset or Juvenile-Onset Still's Disease or Still's Disease, Juvenile Onset or Systemic Arthritis, Juvenile or Arthritis, Juvenile Systemic or Juvenile Systemic Arthritis or Juvenile-Onset Stills Disease or Juvenile Onset Stills Disease or Stills Disease, Juvenile-Onset or Polyarthritis, Juvenile, Rheumatoid Factor Positive or Polyarticular Juvenile Idiopathic Arthritis or Polyarticular-Course Juvenile Idiopathic Arthritis or PCJIA or PJIA Polyarticular Juvenile Idiopathic Arthritis OR Arthritis, Rheumatoid or Rheumatoid Arthritis OR Felty Syndrome or Syndrome, Felty or Felty's Syndrome or Feltys Syndrome or Syndrome, Felty's or Familial Felty's Syndrome or Familial Feltys Syndrome or Felty's Syndrome, Familial or Syndrome, Familial Felty's or Rheumatoid Arthritis, Splenomegaly and Neutropenia or Familial Felty Syndrome or Felty Syndrome, Familial or Syndrome, Familial Felty OR Rheumatoid Vasculitis or Rheumatoid Vasculitides or Vasculitides, Rheumatoid or Vasculitis, Rheumatoid OR Sjogren's Syndrome or Sjogrens Syndrome or Syndrome, Sjogren's or Sjogren Syndrome or Sicca Syndrome or Syndrome, Sicca OR Still's Disease, Adult-Onset or Still's Disease, Adult Onset or Stills Disease, Adult-Onset or Adult-Onset Still's Disease or Adult Onset Still's Disease or Adult-Onset Stills Disease or Still Disease, Adult-Onset or Still Disease, Adult Onset or Adult-Onset Still Disease or Adult Onset Still Disease OR Autoimmune Diseases of the Nervous System or Autoimmune Diseases, Neurologic or Autoimmune Disease, Neurologic or Neurologic Autoimmune Disease or Neurologic Autoimmune Diseases or Autoimmune Disorders, Nervous System or Autoimmune Nervous System Diseases or Nervous System Autoimmune Diseases or Autoimmune Diseases, Nervous System or Autoimmune Disorders of the Nervous System or Autoimmune Encephalitis or Autoimmune Encephalitides or Encephalitis, Autoimmune or Antibody-Mediated Encephalitis or Antibody Mediated Encephalitis or Antibody-Mediated Encephalitides or Encephalitis, Antibody-Mediated or Immune Disorders, Nervous System or Nervous System Immune Diseases or Nervous System Immune Disorders or Immune Diseases, Nervous System OR Anti-N-Methyl-D-Aspartate Receptor Encephalitis or Anti N Methyl D Aspartate Receptor Encephalitis or Anti-N-Methyl-D-Aspartate Receptor Encephalitides or Encephalitides, Anti-N-Methyl-D-Aspartate Receptor or Encephalitis, Anti-N-Methyl-D-Aspartate Receptor or Anti-NMDA Receptor Encephalitis or Anti NMDA Receptor Encephalitis or Anti-NMDA Receptor Encephalitides or Encephalitides, Anti-NMDA Receptor or Encephalitis, Anti-NMDA Receptor or Receptor Encephalitides, Anti-NMDA or Receptor Encephalitis, Anti-NMDA or Anti-NMDAR Encephalitis or Anti NMDAR Encephalitis or Anti-NMDAR Encephalitides or Encephalitides, Anti-NMDAR or Encephalitis, Anti-NMDAR or Non-paraneoplastic Anti-N-Methyl-D-Aspartate Receptor Encephalitis or Non paraneoplastic Anti N Methyl D Aspartate Receptor Encephalitis or Non-paraneoplastic Anti-NMDA Receptor Encephalitis or Non paraneoplastic Anti NMDA Receptor Encephalitis or Non-paraneoplastic Anti-NMDAR Encephalitis or Anti-NMDAR Encephalitides, Non-paraneoplastic or Anti-NMDAR Encephalitis, Non-paraneoplastic or Encephalitides, Non-paraneoplastic Anti-NMDAR or Encephalitis, Non-paraneoplastic Anti-NMDAR or Non paraneoplastic Anti NMDAR Encephalitis or Non-paraneoplastic Anti-NMDAR Encephalitides or Paraneoplastic Anti-N-Methyl-D-Aspartate Receptor Encephalitis or Paraneoplastic Anti N Methyl D Aspartate Receptor Encephalitis or Paraneoplastic Anti-NMDA Receptor Encephalitis or Paraneoplastic Anti NMDA Receptor Encephalitis or Paraneoplastic Anti-NMDAR Encephalitis or Anti-NMDAR Encephalitides, Paraneoplastic or Anti-NMDAR Encephalitis, Paraneoplastic or Encephalitides, Paraneoplastic Anti-NMDAR or Encephalitis, Paraneoplastic Anti-NMDAR or Paraneoplastic Anti NMDAR Encephalitis or Paraneoplastic Anti-NMDAR Encephalitides OR Stiff-Person Syndrome or Stiff Person Syndrome or Syndrome, Stiff-Person or Stiff-Man Syndrome or Stiff Man Syndrome or Syndrome, Stiff-Man or Stiffman Syndrome or Syndrome, Stiffman or Stiff-Baby Syndrome or Stiff-Baby Syndromes or Syndrome, Stiff-Baby or Syndromes, Stiff-Baby or Congenital Stiff-Man Syndrome or Congenital Stiff Man Syndrome or Congenital Stiff-Man Syndromes or Stiff-Man Syndrome, Congenital or Stiff-Man Syndromes, Congenital or Syndrome, Congenital Stiff-Man or Syndromes, Congenital Stiff-Man or Congenital Stiff-Person Syndrome or Congenital Stiff-Person Syndromes or Stiff-Person Syndrome, Congenital or Stiff-Person Syndromes, Congenital or Syndrome, Congenital Stiff-Person or Syndromes, Congenital Stiff-Person or Startle Syndrome or Startle Syndromes or Syndrome, Startle or Syndromes, Startle or Moersch-Woltmann Syndrome or Moersch Woltmann Syndrome or Syndrome, Moersch-Woltmann or Stiff-Trunk Syndrome or Stiff Trunk Syndrome or Stiff-Trunk Syndromes or Syndrome, Stiff-Trunk or Syndromes, Stiff-Trunk or Familial Hyperekplexia or Familial Hyperekplexias or Hyperekplexia, Familial or Hyperekplexias, Familial or Hereditary Hyperekplexia or Hereditary Hyperekplexias or Hyperekplexia, Hereditary or Hyperekplexias, Hereditary OR Uveomeningoencephalitic Syndrome or Syndrome, Uveomeningoencephalitic or Uveomeningoencephalitis or Uveomeningoencephalitides or VKH Syndrome or Syndrome, VKH or Syndrome, VKH (Vogt Koyanagi Harada) or VKH Syndrome (Vogt Koyanagi Harada) or Vogt-Koyanagi-Harada Disease or Disease, Vogt-Koyanagi-Harada or Vogt Koyanagi Harada Disease or VKH (Vogt Koyanagi Harada) Syndrome or Vogt-Koyanagi-Harada Syndrome or Syndrome, Vogt-Koyanagi-Harada or Vogt Koyanagi Harada Syndrome or Syndrome, Vogt Koyanagi Harada OR Autoimmune Hypophysitis or Autoimmune Hypophysitides or Hypophysitides, Autoimmune or Lymphoid Hypophysitis or Hypophysitides, Lymphoid or Hypophysitis, Lymphoid or Lymphoid Hypophysitides or Hypophysitis, Lymphocytic or Hypophysitis, Autoimmune or Lymphocytic Hypophysitis or Hypophysitides, Lymphocytic or Lymphocytic Hypophysitides or Lymphocytic Panhypophysitis or Lymphocytic Panhypophysitides or Panhypophysitides, Lymphocytic or Panhypophysitis, Lymphocytic or Anti-PIT-1 Antibody Syndrome or Anti PIT 1 Antibody Syndrome or Anti-PIT-1 Antibody Syndromes or Antibody Syndrome, Anti-PIT-1 or Antibody Syndromes, Anti-PIT-1 or Syndrome, Anti-PIT-1 Antibody or Syndromes, Anti-PIT-1 Antibody or Idiopathic Granulomatous Hypophysitis or Granulomatous Hypophysitides, Idiopathic or Granulomatous Hypophysitis, Idiopathic or Hypophysitides, Idiopathic Granulomatous or Hypophysitis, Idiopathic Granulomatous or Idiopathic Granulomatous Hypophysitides or IgG4-Related Hypophysitis or Hypophysitides, IgG4-Related or Hypophysitis, IgG4-Related or IgG4 Related Hypophysitis or IgG4-Related Hypophysitides or Lymphocytic Infundibuloneurohypophysitis or Infundibuloneurohypophysitides, Lymphocytic or Infundibuloneurohypophysitis, Lymphocytic or Lymphocytic Infundibuloneurohypophysitides or Lymphocytic Adenohypophysitis or Adenohypophysitides, Lymphocytic or Adenohypophysitis, Lymphocytic or Lymphocytic Adenohypophysitides OR Autoimmune Lymphoproliferative Syndrome or Autoimmune Lymphoproliferative Syndromes or Lymphoproliferative Syndrome, Autoimmune or Lymphoproliferative Syndromes, Autoimmune or Syndrome, Autoimmune Lymphoproliferative or Syndromes, Autoimmune Lymphoproliferative or Canale-Smith Syndrome or Canale-Smith Syndromes or Syndrome, Canale-Smith or Syndromes, Canale-Smith or Autoimmune Lymphoproliferative Syndrome, Type I, Autosomal Dominant or Canale Smith Syndrome or Syndrome, Canale Smith or Autoimmune Lymphoproliferative Syndrome Type 1, Autosomal Dominant or Autoimmune Lymphoproliferative Syndrome Type 2B or Caspase-8 Deficiency or Caspase-8 Deficiencies or Deficiencies, Caspase-8 or Deficiency, Caspase-8 or Autoimmune Lymphoproliferative Syndrome Type 2B (ALPS2B) or Autoimmune Lymphoproliferative Syndrome, Type IIb or Caspase 8 Deficiency or Caspase 8 Deficiencies or Deficiencies, Caspase 8 or Deficiency, Caspase 8 OR Autoimmune Pancreatitis or Autoimmune Pancreatitides or Pancreatitis, Autoimmune or Type 1 Autoimmune Pancreatitis or IgG4-related Pancreatitis or IgG4 related Pancreatitis or IgG4-related Pancreatitides or Pancreatitis, IgG4-related or Type 1 AIP or Type 2 Autoimmune Pancreatitis or Type 2 AIP or Idiopathic Duct-centric Pancreatitis or Duct-centric Pancreatitis, Idiopathic or Idiopathic Duct centric Pancreatitis or Idiopathic Duct-centric Pancreatitides or Pancreatitis, Idiopathic Duct-centric OR Birdshot Chorioretinopathy or Birdshot Chorioretinopathies or Chorioretinopathy, Birdshot or Birdshot Chorioretinitis or Chorioretinitis, Birdshot or Birdshot Retinochoroiditis or Birdshot Retinochoroiditides or Retinochoroiditis, Birdshot or Birdshot Retinochoroidopathy or Birdshot Retinochoroidopathies or Retinochoroidopathy, Birdshot OR Dermatitis Herpetiformis or Duhring's Disease or Disease, Duhring's or Duhrings Disease or Duhring Disease or Disease, Duhring OR Diabetes Mellitus, Type 1 or Diabetes Mellitus, Insulin-Dependent or Diabetes Mellitus, Insulin Dependent or Insulin-Dependent Diabetes Mellitus or Diabetes Mellitus, Juvenile-Onset or Diabetes Mellitus, Juvenile Onset or Juvenile-Onset Diabetes Mellitus or IDDM or Juvenile-Onset Diabetes or Diabetes, Juvenile-Onset or Juvenile Onset Diabetes or Diabetes Mellitus, Sudden-Onset or Diabetes Mellitus, Sudden Onset or Sudden-Onset Diabetes Mellitus or Type 1 Diabetes Mellitus or Diabetes Mellitus, Insulin-Dependent, 1 or Insulin-Dependent Diabetes Mellitus 1 or Insulin Dependent Diabetes Mellitus 1 or Type 1 Diabetes or Diabetes, Type 1 or Diabetes Mellitus, Type I or Diabetes, Autoimmune or Autoimmune Diabetes or Diabetes Mellitus, Brittle or Brittle Diabetes Mellitus or Diabetes Mellitus, Ketosis-Prone or Diabetes Mellitus, Ketosis Prone or Ketosis-Prone Diabetes Mellitus OR Glomerulonephritis, IGA or Glomerulonephritides, IGA or Berger's Disease or Bergers Disease or IGA Glomerulonephritis or Nephropathy, IGA or Iga Nephropathy 1 or Nephropathy 1, Iga or Immunoglobulin A Nephropathy or Nephropathy, Immunoglobulin A or Nephritis, IGA Type or IGA Type Nephritis or Berger Disease or IGA Nephropathy OR Glomerulonephritis, Membranous or Glomerulonephritides, Membranous or Membranous Glomerulonephritides or Membranous Glomerulonephritis or Nephropathy, Membranous or Membranous Glomerulopathy or Glomerulopathy, Membranous or Membranous Nephropathy or Extramembranous Glomerulopathy or Glomerulopathy, Extramembranous or Membranous Glomerulonephropathy or Glomerulonephropathy, Membranous or Heymann Nephritis or Nephritis, Heymann or Idiopathic Membranous Glomerulonephritis or Glomerulonephritides, Idiopathic Membranous or Glomerulonephritis, Idiopathic Membranous or Idiopathic Membranous Glomerulonephritides or Membranous Glomerulonephritides, Idiopathic or Membranous Glomerulonephritis, Idiopathic or Idiopathic Membranous Nephropathy or Membranous Nephropathy, Idiopathic or Nephropathy, Idiopathic Membranous OR Graves Disease or Disease, Graves or Basedow Disease or Disease, Basedow or Graves' Disease or Disease, Graves' or Exophthalmic Goiter or Exophthalmic Goiters or Goiters, Exophthalmic or Goiter, Exophthalmic or Hyperthyroidism, Autoimmune or Basedow's Disease or Basedows Disease or Disease, Basedow's OR Graves Ophthalmopathy or Ophthalmopathy, Graves or Ophthalmopathies, Thyroid-Associated or Thyroid-Associated Ophthalmopathies or Thyroid Associated Ophthalmopathies or Thyroid Eye Disease or Disease, Thyroid Eye or Eye Disease, Thyroid or Thyroid Eye Diseases or Thyroid-Associated Ophthalmopathy or Thyroid Associated Ophthalmopathy or Dysthyroid Ophthalmopathy or Dysthyroid Ophthalmopathies or Ophthalmopathy, Dysthyroid or Graves Eye Disease or Disease, Graves Eye or Eye Disease, Graves or Graves Orbitopathy or Orbitopathy, Graves or Ophthalmopathy, Thyroid-Associated or Ophthalmopathy, Thyroid Associated or Myopathic Ophthalmopathy or Myopathic Ophthalmopathies or Ophthalmopathy, Myopathic or Congestive Ophthalmopathy or Congestive Ophthalmopathies or Ophthalmopathy, Congestive or Edematous Ophthalmopathy or Edematous Ophthalmopathies or Ophthalmopathy, Edematous or Ophthalmopathy, Infiltrative or Infiltrative Ophthalmopathies or Infiltrative Ophthalmopathy OR Hepatitis, Autoimmune or Autoimmune Hepatitides or Hepatitides, Autoimmune or Autoimmune Chronic Hepatitis or Autoimmune Chronic Hepatitides or Chronic Hepatitides, Autoimmune or Chronic Hepatitis, Autoimmune or Hepatitides, Autoimmune Chronic or Hepatitis, Autoimmune Chronic or Autoimmune Hepatitis OR Latent Autoimmune Diabetes in Adults or Diabetes Mellitus Type 1.5 or Type 1.5 Diabetes Mellitus or Type 1.5 Diabetes or Diabetes, Type 1.5 or LADA, Latent Autoimmune Diabetes in Adults or Latent Autoimmune Diabetes of Adults OR Linear IgA Bullous Dermatosis or Linear IgA Dermatosis or Dermatoses, Linear IgA or Dermatosis, Linear IgA or IgA Dermatoses, Linear or IgA Dermatosis, Linear or Linear IgA Dermatoses or Drug-induced Linear IgA Bullous Dermatosis or Drug induced Linear IgA Bullous Dermatosis or Drug-induced Linear IgA Dermatosis or Drug induced Linear IgA Dermatosis or Linear IgA IgG Bullous Dermatosis or Linear IgA IgG Dermatosis or Chronic Bullous Disease of Childhood OR Lupus Erythematosus, Systemic or Systemic Lupus Erythematosus or Lupus Erythematosus Disseminatus or Libman-Sacks Disease or Disease, Libman-Sacks or Libman Sacks Disease OR Lupus Nephritis or Lupus Glomerulonephritis or Nephritis, Lupus or Lupus Nephritides or Nephritides, Lupus or Glomerulonephritis, Lupus or Glomerulonephritides, Lupus or Lupus Glomerulonephritides OR Lupus Vasculitis, Central Nervous System or Central Nervous System Lupus Vasculitis or Systemic Lupus Erythematosis, Central Nervous System or Central Nervous System Lupus or Central Nervous System Systemic Lupus Erythematosis or Neuropsychiatric Systemic Lupus Erythematosus or Lupus Meningoencephalitis or Lupus Meningoencephalitides or Meningoencephalitides, Lupus or Meningoencephalitis, Lupus OR Ophthalmia, Sympathetic or Ophthalmias, Sympathetic or Sympathetic Ophthalmia or Sympathetic Ophthalmias or Uveitis, Sympathetic or Sympathetic Uveitides or Sympathetic Uveitis or Uveitides, Sympathetic OR Pemphigoid, Bullous or Bullous Pemphigoid or Pemphigoid or Pemphigoids OR Pemphigus or Pemphigus Vulgaris or Pemphigus Foliaceus or Foliaceus, Pemphigus OR Polyendocrinopathies, Autoimmune or Autoimmune Polyendocrinopathy or Polyendocrinopathy, Autoimmune or Polyglandular Type III Autoimmune Syndrome or Polyglandular Autoimmune Syndrome, Type 3 or Autoimmune Syndrome Type III, Polyglandular or Autoimmune Polyglandular Syndrome Type III or Autoimmune Polyglandular Syndrome, Type 3 or Polyglandular Type I Autoimmune Syndrome or APECED or APS Type 1 or Autoimmune Polyendocrinopathy Syndrome Type 1 or Autoimmune Polyendocrinopathy with Candidiasis and Ectodermal Dystrophy or Autoimmune Polyendocrinopathy-Candidiasis-Ectodermal Dystrophy or Autoimmune Polyendocrinopathy Candidiasis Ectodermal Dystrophy or Polyendocrinopathy-Candidiasis-Ectodermal Dystrophy, Autoimmune or Autoimmune Polyendocrinopathy-Candidiasis-Ectodermal-Dystrophy or Autoimmune Polyglandular Syndrome Type I or Autoimmune Polyglandular Syndrome, Type 1 or Autoimmune Polyglandular Syndrome, Type I or Autoimmune Syndrome Type I, Polyglandular or Polyendocrinopathy-Candidiasis-Ectodermal-Dystrophy, Autoimmune or Polyendocrinopathy Candidiasis Ectodermal Dystrophy, Autoimmune or Polyglandular Autoimmune Syndrome, Type 1 or Polyglandular Autoimmune Syndrome, Type I or AIRE Deficiency or AIRE Deficiencies or Deficiency, AIRE or Polyglandular Type II Autoimmune Syndrome or Autoimmune Polyendocrine Syndrome, Type II or Autoimmune Polyglandular Syndrome Type II or Autoimmune Syndrome Type II, Polyglandular or Diabetes Mellitus, Addison Disease, Myxedema or Schmidt's Syndrome or Syndrome, Schmidt's or Multiple Endocrine Deficiency Syndrome, Type 2 or Polyendocrine Autoimmune Syndrome, Type II or Polyglandular Autoimmune Syndrome, Type 2 or Polyglandular Deficiency Syndrome, Type 2 or Schmidt Syndrome or Syndrome, Schmidt or Autoimmune Polyendocrine Syndrome, Type 2 or Diabetes Mellitus, Addison's Disease, Myxedema OR Purpura, Thrombocytopenic, Idiopathic or Werlhof's Disease or Disease, Werlhof's or Werlhofs Disease or Autoimmune Thrombocytopenic Purpura or Autoimmune Thrombocytopenic Purpuras or Purpura, Autoimmune Thrombocytopenic or Purpuras, Autoimmune Thrombocytopenic or Purpura, Thrombocytopenic, Autoimmune or Immune Thrombocytopenia or Immune Thrombocytopenias or Thrombocytopenia, Immune or Thrombocytopenias, Immune or Autoimmune Thrombocytopenia or Autoimmune Thrombocytopenias or Thrombocytopenia, Autoimmune or Thrombocytopenias, Autoimmune or Immune Thrombocytopenic Purpura or Immune Thrombocytopenic Purpuras or Purpura, Immune Thrombocytopenic or Purpuras, Immune Thrombocytopenic or Thrombocytopenic Purpura, Immune or Thrombocytopenic Purpuras, Immune or Thrombocytopenic Purpura, Autoimmune or Werlhof Disease or Disease, Werlhof or Idiopathic Thrombocytopenic Purpura or Idiopathic Thrombocytopenic Purpuras or Purpura, Idiopathic Thrombocytopenic or Purpuras, Idiopathic Thrombocytopenic or Thrombocytopenic Purpura, Idiopathic or Thrombocytopenic Purpuras, Idiopathic OR Thyroiditis, Autoimmune or Autoimmune Thyroiditides or Thyroiditides, Autoimmune or Autoimmune Thyroiditis or Thyroiditis, Lymphocytic or Lymphocytic Thyroiditides or Lymphocytic Thyroiditis or Thyroiditides, Lymphocytic or Thyroiditis, Lymphomatous or Lymphomatous Thyroiditides or Lymphomatous Thyroiditis or Thyroiditides, Lymphomatous OR Hashimoto Disease or Disease, Hashimoto or Chronic Lymphocytic Thyroiditis or Chronic Lymphocytic Thyroiditides or Lymphocytic Thyroiditides, Chronic or Lymphocytic Thyroiditis, Chronic or Thyroiditides, Chronic Lymphocytic or Thyroiditis, Chronic Lymphocytic or Hashimoto Struma or Hashimoto's Struma or Hashimoto's Syndrome or Hashimoto Syndrome or Hashimoto's Syndromes or Hashimotos Syndrome or Syndrome, Hashimoto's or Syndromes, Hashimoto's or Hashimoto's Disease or Disease, Hashimoto's or Hashimotos Disease or Hashimoto Thyroiditis or Hashimoto Thyroiditides or Thyroiditides, Hashimoto or Thyroiditis, Hashimoto OR Postpartum Thyroiditis or Postpartum Thyroiditides or Thyroiditides, Postpartum or Thyroiditis, Postpartum or Post-partum Thyroiditis or Post partum Thyroiditis or Post-partum Thyroiditides or Thyroiditides, Post-partum or Thyroiditis, Post-partum OR Undifferentiated Connective Tissue Diseases or Undifferentiated Connective Tissue Disease OR Demyelinating Autoimmune Diseases, CNS or Demyelinating Autoimmune Disorders, CNS or Demyelinating Disease, Autoimmune, CNS or CNS Demyelinating Autoimmune Diseases or Autoimmune Demyelinating Diseases, CNS or Autoimmune Demyelinating Disorders, CNS or CNS Autoimmune Demyelinating Disorders or Demyelinating Autoimmune Diseases, Central Nervous System or Autoimmune Demyelinating Diseases, Central Nervous System or Autoimmune Diseases, Demyelinating, Brain or Demyelinating Autoimmune Diseases, Brain or Brain Autoimmune Demyelinating Diseases or Spinal Cord Demyelinating Autoimmune Diseases or Autoimmune Demyelinating Diseases, Spinal Cord or Demyelinating Autoimmune Diseases, Spinal Cord or Autoimmune Demyelinating Diseases, Cerebral or Demyelinating Autoimmune Diseases, Cerebral or Cerebral Demyelinating Diseases, Autoimmune OR Myasthenia Gravis or Myasthenia Gravis, Ocular or Ocular Myasthenia Gravis or Myasthenia Gravis, Generalized or Generalized Myasthenia Gravis or Muscle-Specific Receptor Tyrosine Kinase Myasthenia Gravis or Muscle Specific Receptor Tyrosine Kinase Myasthenia Gravis or Muscle-Specific Tyrosine Kinase Antibody Positive Myasthenia Gravis or Muscle Specific Tyrosine Kinase Antibody Positive Myasthenia Gravis or MuSK MG or MuSK Myasthenia Gravis or Myasthenia Gravis, MuSK or Anti-MuSK Myasthenia Gravis or Anti MuSK Myasthenia Gravis or Myasthenia Gravis, Anti-MuSK OR Nervous System Autoimmune Disease, Experimental or Disease Models, Autoimmune, Nervous System or Nervous System Autoimmune Disease Models or Autoimmune Disease Models, Nervous System or Autoimmune Myositis, Experimental or Myositis, Autoimmune Experimental or Autoimmune Experimental Myositis or Experimental Myositis, Autoimmune or Myositis, Experimental Autoimmune or Experimental Autoimmune Myositis OR Polyradiculoneuropathy or Polyradiculoneuropathies or Polyradiculoneuritis or Polyradiculoneuritides or Autoimmune Demyelinating Disease, Peripheral or Demyelinating Disease, Peripheral Autoimmune or Peripheral Autoimmune Demyelinating Disease or Demyelinating Autoimmune Disease, Peripheral

#2=Subdoligranulum variabile OR subdoligranulum
